# Macular Telangiectasia Type 2: The Role of Optical Coherence Tomography and Management Options

**DOI:** 10.3390/jcm15041327

**Published:** 2026-02-07

**Authors:** David-Ionuț Beuran, Ioana Ruxandra Boca, Cătălin Cornăcel, Călin Petru Tătaru, Cătălina Ioana Tătaru, Maria-Emilia Cerghedean-Florea, Cosmin Adrian Teodoru

**Affiliations:** 1 Doctoral School, “Carol Davila” University of Medicine and Pharmacy, 020021 Bucharest, Romania; 2 Ophthalmology Department, “Dr. Carol Davila” Central Military Emergency University Hospital, 010825 Bucharest, Romania; 3 Ophthalmology Department, “Carol Davila” University of Medicine and Pharmacy, 020021 Bucharest, Romania; 4 Department of Ophthalmology, Clinical Hospital for Ophthalmological Emergencies, 010464 Bucharest, Romania; 5 Faculty of Medicine, “Lucian Blaga” University of Sibiu, 550024 Sibiu, Romania

**Keywords:** macular telangiectasia type 2, optical coherence tomography, OCT biomarkers, non-proliferative mactel, proliferative mactel, ciliary neurotrophic factor, anti-VEGF therapy

## Abstract

**Background/Objectives**: Macular Telangiectasia Type 2 (MacTel type 2) is a rare, progressive retinal disease that can lead to central vision loss. Optical coherence tomography (OCT) plays a crucial role in the early diagnosis, monitoring, and prognostic assessment of this condition. This narrative review aims to summarize the clinical features, OCT findings, and current management strategies for MacTel type 2. **Methods**: A literature search of PubMed, MEDLINE, and Google Scholar was performed for articles published from October 1993 to September 2025 using keywords related to MacTel type 2, OCT, clinical features, and treatment. All relevant clinical studies, including observational studies, clinical trials, and case series, were considered. The literature was screened independently by two authors, and a total of 69 articles were included. **Results**: Characteristic OCT findings include foveal cavitation, hyperreflective middle retinal layers, inner and outer retinal cavities, ellipsoid zone disruption, and retinal pigment clumps. Central macular thickness is consistently reduced, and structural biomarkers identified on OCT correlate with visual acuity decline. Treatment strategies vary by disease stage: non-proliferative MacTel type 2 currently has no universally effective therapy, although neuroprotective interventions such as ciliary neurotrophic factor (CNTF) show promising results. Proliferative MacTel type 2 is primarily managed with anti-vascular endothelial growth factor therapy (anti-VEGF), demonstrating functional and anatomical improvements. **Conclusions**: OCT provides essential structural evaluation for monitoring MacTel type 2, while treatment approaches remain stage-dependent. Emerging therapies, including CNTF implants and novel anti-VEGF agents, hold potential for improving outcomes.

## 1. Introduction

Macular Telangiectasia Type 2 (MacTel Type 2) is a bilateral, progressive retinal disorder characterized by parafoveal telangiectatic capillary changes and neurosensory retinal atrophy, leading to gradual central vision impairment [1,2,3]. This rare condition has a reported prevalence ranging from 0.02% to 0.1% and primarily affects older adults, with a mean age of 62–65 years. A slight female predominance has been observed in some populations, although the disease is likely underdiagnosed due to the limitations of fundus photography-based detection [4,5,6].

Patients often experience blurred vision, paracentral scotomas, metamorphopsia, or difficulty reading, while fundus examination can reveal reduced retinal transparency, crystalline deposits, telangiectatic capillaries, and pigment hypertrophy [7]. Multimodal imaging, including fluorescein angiography and optical coherence tomography (OCT), demonstrates telangiectatic vessels, intraretinal cavities, photoreceptor disruption, and retinal thinning, reflecting both vascular and neurodegenerative processes [2,3].

Recent studies have clarified the multifactorial pathophysiology of MacTel Type 2. Variants in phosphoglycerate dehydrogenase (PHGDH) and phosphoserine aminotransferase 1 (PSAT1) reduce retinal serine and glycine levels, leading to the accumulation of toxic deoxysphingolipids that disrupt Müller glial cells and promote photoreceptor degeneration. In parallel, common variants affecting retinal vascular architecture act independently, converging with metabolic dysregulation to drive photoreceptor loss and disease progression [8,9,10].

The objective of this narrative review is to summarize the current knowledge on MacTel Type 2, with a particular focus on the role of OCT in diagnosis and monitoring, and stage-dependent management strategies. By providing an updated synthesis of available evidence, this review aims to guide clinicians in early detection, monitoring, and treatment decision-making, while highlighting areas for future research.

## 2. Materials and Methods

A narrative review of the literature on MacTel type 2 was conducted to summarize clinical features, OCT findings, and management options. A systematic search of PubMed, MEDLINE, and Google Scholar was performed for articles published from October 1993 to September 2025. The search used the following keywords: “Macular Telangiectasia type 2” or “MacTel type 2” combined with “classification”, “epidemiology”, “genetics”, “pathogenesis” “clinical features”, “optical coherence tomography”, “central retinal thickness”, “multimodal imaging”, “paraclinical features”, “differential diagnosis”, “associations”, “treatment”, “carbonic anhydrase inhibitors”, “carotenoids”. “laser photocoagulation”, “photodynamic therapy”, “transpupillary thermotherapy”, “intravitreal anti-VEGF”, “ciliary neurotrophic factor”, “pars plana vitrectomy”, “future studies”, and “prognosis”.

All relevant clinical studies, including observational studies, clinical trials, and case series, were considered. Exclusion criteria included non-human studies and articles lacking clinical or imaging data relevant to MacTel type 2. The literature was screened independently by two authors.

A total of 69 articles were included. Data were extracted on clinical features, characteristic OCT findings, treatment strategies for non-proliferative and proliferative disease, treatment outcomes, and future studies.

Artificial intelligence tools were used solely for paraphrasing and language refinement of the manuscript. These tools did not contribute to data collection, analysis, interpretation of results, or scientific conclusions (ChatGPT, OpenAI, San Francisco, CA, USA; GPT-5.2).

## 3. Literature Review

### 3.1. Classification

Gass and Blodi classified IJFT into three main types of telangiectasia: type 1 includes unilateral aneurysmal telangiectasia; type 2 includes bilateral perifoveal telangiectasia, and type 3 includes capillary obstruction and occlusion. Furthermore, each type was subdivided as follows: type 1A includes unilateral juxtafoveal telangiectasia mostly seen in men, which suggests that it may be a form of Coats disease; type 1B is similar to type 1A, but the telangiectatic alterations are less extended; type 2A is the most common group which is based on bilaterality and no sex predilection; type 2B includes a single case of two brothers with juvenile familial idiopathic retinal telangiectasias with bilateral subretinal neovascularization; type 3A presents important capillary occlusion and type 3B includes similar retinal manifestations but in association with central nervous system impairment [2].

To simplify this classification, Yannuzi and co-workers proposed a new terminology for IJFT, namely idiopathic macular telangiectasia, which was classified into two main types. Type 1 macular telangiectasia (aneurysmal telangiectasia) presents mostly in men, unilaterally, with the often development of macular edema. Type 2 macular telangiectasia (perifoveal telangiectasia) is classified either in a non-proliferative stage characterized by exudative changes and macular atrophy or in a proliferative stage marked by subretinal neovascularization and fibrosis. Occlusive telangiectasia, which represents type 3, was not included in this classification due to the small number of cases. This simplified classification emphasizes clinically relevant features and disease stages, improving reproducibility and practical applicability compared with earlier systems, partly by incorporating findings from optical coherence tomography (OCT) to better define macular changes [11,12].

### 3.2. Epidemiology

MacTel type 2 is a rare retinal disorder that occurs primarily after the fifth decade of life. The Beaver Dam Eye Study reported a prevalence of 0.1% of the disease among the cohort of individuals aged 43 to 86 years of life [4]. Moreover, a 2023 research project involving 1806 patients with MacTel type 2 reported only 2% of cases with age under 40, emphasizing that its development before the fourth decade of life is highly unlikely [13].

Regarding gender and race predilection, a higher tendency for Caucasian women to be affected has been observed in the literature. The MacTel Project Report No. 2 revealed that 81% of the participants were Caucasians, of which 64% were females [14], and the MacTel Project Report Number 10 contained a total of 1733 participants, of which 1046 were females [15]. A different study published in 2022, which analyzed 108 patients with MacTel type 2, revealed a 64% female predominance, which highlights the tendency for women to be affected [16].

### 3.3. Clinical Features

**Figure 1:**
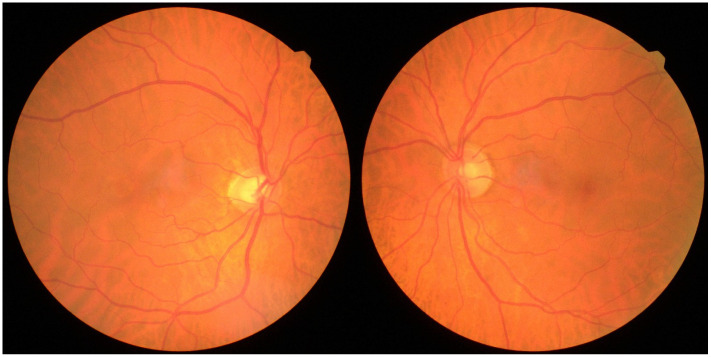
Fundus photo of a patient with early MacTel Type 2 changes, showing a subtle grayish area temporal to the fovea in the right eye. Cup-to-disc ratio asymmetry is noted. OCT demonstrated bilateral features. Images correspond with scans A and B from Figure 2.

Gass and Blodi’s clinical classification of Macular Telangiectasia Type 2 describes five progressive stages: Stage 1, subtle temporal juxtafoveal transparency loss (Figure 1); Stage 2, generalized parafoveal transparency reduction with possible crystalline deposits; Stage 3, right-angled venules; Stage 4, hyperplastic retinal pigment epithelium; and Stage 5, subretinal neovascularization, reflecting advanced disease and visual compromise [2].

Bilateral involvement is characteristic, but sometimes with asymmetric evolution between the eyes. The most incipient sign seen on the fundus exam is the loss of retinal transparency temporally in the macula with the appearance of a greyish retinal area (Figure 1). In the later stages, dilated venules may appear to make a right-angle turn as they insert into deeper retinal layers. Moreover, a retrospective analysis involving 270 eyes from 146 patients highlighted the characteristics of these right-angled vessels (RAV), demonstrating that both retinal arteries and veins can show right-angled dipping. Furthermore, different RAV features may indicate various stages of MacTel type 2; in particular, the presence of a right-angled artery may suggest an underlying proliferative disease process [17].

Additionally, the retinal pigment epithelium may be affected by intraretinal pigment migration, hyperplasia, or atrophy. Another important foveal change that may occur is either a lamellar hole or a full-thickness macular hole. If the disease progresses to the proliferative stage, choroidal neovascularization occurs, and it can be accompanied by lipid exudates and subretinal hemorrhage. These can lead to a final disciform scar if left untreated [3,18,19].

Although MacTel type 2 is conventionally described as a bilateral disease, several reports have documented instances of asymmetric manifestation or, more rarely, unilateral presentation. A recent study (2023) involving 140 patients with MacTel type 2 demonstrated that 60% exhibited symmetrical disease stages in both eyes, whereas 40% displayed asymmetrical progression. Notably, the study identified 12 eyes without detectable alterations on OCT, optical coherence tomography angiography (OCTA), or fluorescein angiography (FA), which were subsequently classified as unilateral cases of MacTel type 2 [20].

Patients usually complain of blurred vision, manifested mainly in near vision rather than in distance vision, along with metamorphopsia. The decrease in visual acuity might be mild in the early stages of the disease, but it usually worsens as progression occurs. Moreover, microperimetry can highlight paracentral microscotomas, which explains the reduced ability of patients to read [3,18].

### 3.4. Optical Coherence Tomography

#### 3.4.1. Typical Changes

**Figure 2:**
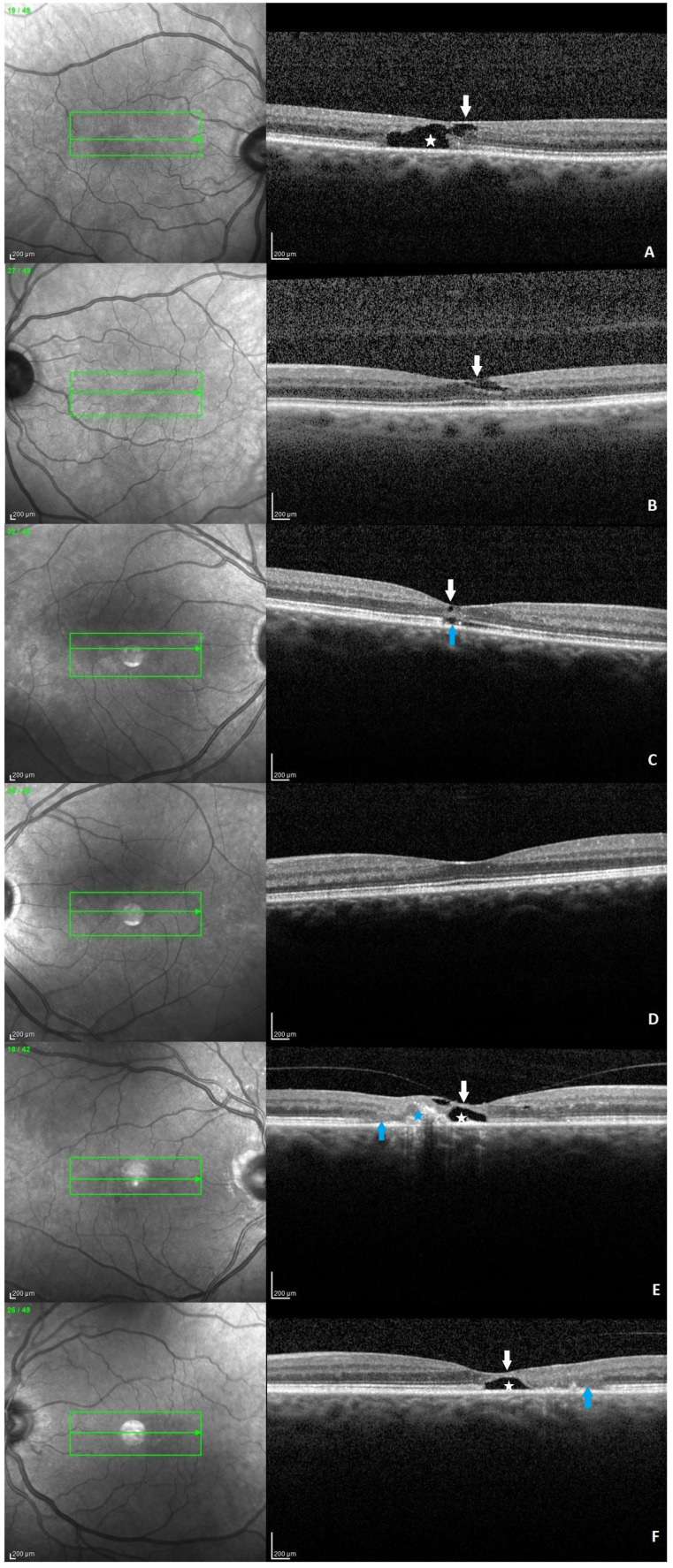
Six spectral-domain OCT B-scans with different features. (**A**) Hypo-reflective inner and outer retinal cavitations (white star) with an ILM drape sign (white arrow). (**B**) Hypo-reflective inner retinal cavity with an ILM drape sign (white arrow). (**C**) Small inner (white arrow) and outer retinal cavitations and disruption of the ellipsoid zone (blue arrow). (**D**) Flattening of the foveal contour. (**E**) Hypo-reflective inner and outer retinal cavities (white star), ILM drage sign (white arrow), retinal pigment clumps (blue star), discontinuity of the ellipsoid zone with outer retinal layer atrophy (blue arrow). (**F**) Hypo-reflective outer retinal cavity (white star) with ILM drape sign (white arrow), discontinuity of the ellipsoid zone with outer retinal layer atrophy (blue arrow). Images (**A**,**B**) correspond to the previously presented fundus photos.

OCT provides valuable information for the diagnosis and monitoring of patients with MacTel type 2. In a 2022 study, Venkatesh et al. examined 212 eyes from 108 patients and reported characteristic OCT features. The following changes were described starting from the inner to the outer retinal layers: (a) irregular foveal contour, (b) internal limiting membrane (ILM) drape, (c) hyper-reflectivity of the middle retinal layers, (d) superficial hyper-reflective retinal dots-retinal crystals, (e) hypo-reflective inner retinal cavities, (f) hypo-reflective outer retinal cavities, (g) outward bending of inner retinal layers, (h) hyper-reflective retinal pigment clumps, (i) subfoveal subretinal fluid, (j) pseudohole, lamellar macular hole or full-thickness macular hole, (k) subretinal neovascular membrane, (l) retinochoroidal anastomosis (RCA) (Figure 2). Furthermore, in the same study, the most frequent and early-onset feature observed was the presence of hyper-reflective middle retinal layers (87%). The second most common finding was represented by hypo-reflective inner retinal cavities (72.9%) [16].

Another study from 2020 that included 129 patients from Korea, found that the most common change seen on OCT was the presence of hypo-reflective cavities (77.7%), followed by discontinuity of the interdigitation zone (56.6%), ellipsoid zone (52.4%), and external limiting membrane (40.4%) [21]. In a study from 2021, Peto et al. described ellipsoid zone break in 602 eyes (61.8%) and outer retina cavities in 243 eyes (24.9%) from a number of 974 eyes with available OCT [22] (Table 1).

Other common features encountered were ILM drape, hypo-reflective outer retinal cavities, outward bending of inner retinal layers without subfoveal subretinal fluid, and irregularity of foveal contour, along with subretinal neovascular membrane and retinochoroidal anastomosis [23].

#### 3.4.2. Central Retinal Thickness

Central macular thickness (CMT) is significantly reduced in MacTel type 2 compared with healthy controls, with values of 214.1 µm versus 258.2 µm (*p* < 0.0001) for central retinal thickness and 279.6 µm versus 323.3 µm (*p* < 0.0001) for parafoveal thickness, indicating characteristic structural thinning [24].

In a separate cohort, CMT was also lower in MacTel type 2 compared with controls (243.6 µm vs. 263.6 µm, *p* = 0.0001), with central choroidal thickness reduced as well (*p* = 0.014), supporting macular thinning as a consistent biomarker of the disease [25].

#### 3.4.3. Clinical Correlations

EZ continuity on OCT reflects photoreceptor integrity, and its disruption correlates with functional visual deficits. Peto et al. described the associations between visual acuity loss and the location of EZ loss reflectivity in MacTel type 2 patients. Participants without EZ loss at baseline lost −0.93 letters/year, those with non-central EZ loss lost −1.09 letters/year (*p* = 0.18 compared with no EZ loss present at baseline), and those with central EZ loss lost −1.40 letters/year (*p* < 0.001 compared with no EZ loss present at baseline) over a mean follow-up period of 4.2 ± 1.6 years [22]. In another study, including 56 eyes of 31 participants diagnosed with MacTel type 2, Hereen et al. described that the progression of EZ loss was strongly associated with the progression of relative and absolute scotomas, but not with loss in best corrected visual acuity. They did not evaluate the positions of EZ in relation to the fovea [26].

Hyporeflective intraretinal spaces, also known as cavitations, are known to fluctuate over time. A 2020 study tracked their progression over two years in a MacTel type 2 cohort (51 eyes from 51 patients) using OCT scans from sham-treated eyes in a phase II clinical trial. The researchers observed that cavitation volume increased slowly but consistently and was strongly associated with poorer visual acuity, independent of EZ loss. Cavitation volume also showed no correlation with EZ loss, suggesting that the two may represent distinct pathological processes. Overall, the findings support cavitation volume as a valuable biomarker for monitoring disease progression in MacTel type 2 [27].

Clemont et al. described the following six MacTel type 2 characteristics based on OCT: apparent foveal detachment, EZ break, hyperreflectivity or loss of the outer nuclear layer, intra-retinal pigment migration, or subretinal neovascularization/fibrosis. Eyes exhibiting at least one of these patterns showed significantly reduced mean visual acuity compared to eyes not in the spectrum [14]. Venkatesh et al. described that hyperreflective middle retinal layers, inner and outer retinal cavities, and ILM drape were associated with poor vision in the non-proliferative group, and the presence of retinal pigment clumps, subretinal fluid, and foveal contour irregularity were associated with poor vision in the proliferative group [16].

### 3.5. Other Paraclinical Features

#### 3.5.1. Fluorescein Angiography

The classification of the disease was primarily based on the changes observed on FA, which were among the most significant changes leading to Gass and Blodi, and Yanuzzi’s classifications of MacTel type 2 [1,2]. The characteristic finding is the presence of telangiectatic capillaries, which cause leakage situated temporally to the fovea in the early phase and diffuse hyperfluorescence in the late phase [3,18].

#### 3.5.2. Optical Coherence Tomography Angiography

OCTA is an important non-invasive tool that offers information on retinal and choroidal circulation. Alterations observed in MacTel type 2 include enlargement of the foveal avascular zone (FAZ), increased intercapillary spacing, capillary dropout, telangiectatic changes, and the development of superficial and deep capillary plexus anastomoses. A valuable feature is that OCTA has demonstrated the ability to identify newly developed retinal–choroidal anastomoses without accompanying focal pigmentation, structural fluid exudation, or prior subretinal neovascularization [28,29].

A study from 2016 graded the vascular anomalies discovered among MacTel type 2 patients on the angiography in 4 grades as follows: (1) Deep and/or superficial plexus vascular anomalies temporal to the fovea; (2) Deep and/or superficial plexus vascular anomalies temporal and nasal to the fovea; (3) Diffuse circumferential deep and superficial plexus vascular anomalies; (4) Neovascularization situated in the outer retina [24].

Additionally, OCT-A can detect microvascular alterations that may be visible before appearing on OCT scans. An observational study identified 4 individuals among 630 MacTel patients with typical disease features in one eye, while the fellow eye appeared normal on color fundus photography and SD-OCT. Using OCT-A, all four fellow eyes showed telangiectasia and foveal avascular zone changes in capillary plexuses, despite no visible changes on OCT [30].

#### 3.5.3. Fundus Autofluorescence (FAF)

FAF can be an important tool for evaluating patients with MacTel type 2. A study that included 807 eyes of 420 patients discovered fundus autofluorescence changes in 98.3% of the cases, which were mostly located temporally to the fovea. FAF patterns that were observed included increased FAF, decreased or mixed FAF, and vascular abnormalities [31].

### 3.6. Differential Diagnosis

The differential diagnosis of MacTel type 2 is made with retinal vascular diseases that may cause capillary changes, such as diabetic retinopathy, retinal vein or branch occlusion, and radiation retinopathy. Moreover, the proliferative stage of MacTel type 2, characterized by the formation of subretinal neovascularization, can mimic the exudative type of age-related macular degeneration. In the final stages of both diseases, with the presence of disciform scars, it can be difficult to distinguish between them [7,18].

Important and specific features that may be useful in differentiating MacTel type 2 from other disorders are the characteristic retinal cavities seen on OCT and the bilateral temporal predilection of the disease, which does not respect the horizontal raphe [7]. Furthermore, the intraretinal hypo-reflective spaces do not correspond with an increased retinal thickness, which suggests that these cavities are not filled with fluid, such as those found in diabetic macular edema or cystoid macular edema caused by other disorders. Moreover, the foveal thickness in MacTel type 2 patients is usually decreased [32].

### 3.7. Association with Systemic Diseases

The two systemic diseases that were most frequently associated with MacTel type 2 were diabetes mellitus and hypertension. A study that included 206 eyes of 103 patients with MacTel type 2 showed that almost half of the patients (49%) had diabetes mellitus type 2 and hypertension (46%) [33]. In a similar study, which included 310 participants, a history of hypertension was found in 52% of them, while diabetes mellitus was found in 28% of cases. Moreover, 11% had a history of coronary artery disease, and 43% were obese [14].

Furthermore, other studies found that a history of diabetes mellitus was present in 75%, respectively, 69% of the MacTel type 2 patients [16,20].

It is believed that diabetes and macular telangiectasia may share a common pathway, namely due to Muller cell involvement. Dysfunction of these cells plays an important role in the development of both diseases [33].

In addition, a 2024 study aimed to investigate how the evolution of inner retinal layer thickness in MacTel type 2 patients—with and without diabetes—compares with that of diabetic patients without retinopathy. The study evaluated and compared nerve fiber layer and ganglion cell layer thickness across the full ETDRS grid and within the inner temporal region. MacTel type 2 patients showed inner retinal thinning like that observed in diabetic individuals without retinopathy, and this thinning was not influenced by diabetes status, highlighting the need for further research to clarify its clinical significance [34].

### 3.8. Treatment

#### 3.8.1. Non-Proliferative MacTel Type 2

The treatment modalities differ depending on the stage of the disease. The following options for the non-proliferative phase have been described in different studies: use of carbonic anhydrase inhibitors, use of carotenoids, focal laser photocoagulation, photodynamic therapy, intravitreal injections with anti-vascular endothelial growth factor drugs or steroids, surgical implant with release of ciliary neurotrophic factor, and pars plana vitrectomy with internal limiting membrane peeling (Table 2). Recent clinical trial results with CNTF are encouraging, making it a promising treatment for the non-proliferative stage.

CNTF is a neuroprotective cytokine that preserves photoreceptor structure and function in experimental models resembling MacTel type 2, without affecting associated vascular abnormalities [35,36]. Direct retinal delivery is limited by the blood–retinal barrier; to overcome this, the NT-501 implant, an encapsulated cell-based intravitreal device, was developed. It contains human retinal pigment epithelial (RPE) cells transfected with the CNTF gene, enclosed within a semipermeable membrane that allows CNTF diffusion into the vitreous while protecting the cells from immune attack, providing sustained long-term delivery with minimal systemic exposure and the option for surgical explantation [37].

In a phase 1 trial, seven adults with bilateral MacTel type 2 (48–67 years; 2 men and 5 women; predominantly Caucasian) met eligibility criteria of best corrected visual acuity ≥20/50, no prior intraocular surgery, and ellipsoid zone disruption on OCT (study eye: worse-seeing eye). Over 36 months, participants tolerated NT-501 implants, with stable visual acuity, retinal structure, and retinal sensitivity, and OCT provided important biomarkers for monitoring treatment effects [36]. In a phase 2, multicenter, randomized, single-masked trial, 67 adults with bilateral MacTel type 2 (mean age 62 ± 8.9 years; 61% women; 86% Caucasian) with ellipsoid zone disruption on OCT and baseline best corrected visual acuity around 20/30 were enrolled (99 study eyes). Over 24 months, CNTF-treated eyes showed 31% less photoreceptor loss compared with sham-treated eyes, with better-preserved retinal sensitivity and reading speed [38]. In phase 3 trials NTMT-03-A (58 NT-501, 57 sham) and NTMT-03-B (59 NT-501, 54 sham), patients with bilateral MacTel type 2 received NT-501 implants, or sham. The primary endpoint was ellipsoid zone area loss over 24 months, monitored by OCT. NT-501 slowed EZ loss compared with sham (NTMT-03-A: 0.075 vs. 0.166 mm^2^, *p* < 0.001; NTMT-03-B: 0.111 vs. 0.160 mm^2^, *p* = 0.02), while best-corrected visual acuity and patient-reported visual function were generally preserved. Retinal sensitivity and reading speed showed variable changes between groups [39].

These results, together with the favorable safety profile—mostly mild to moderate adverse events—led to the US FDA approval of Revakinagene taroretcel (ENCELTO™) in March 2025, marking the first approved therapy for MacTel type 2. The neuroprotective benefits outweigh the observed risks [40,41,42].

**Table 2:** Different treatment options and their outcomes for non-proliferative MacTel Type 2.

Treatment	Drug/Compound	Study	Type	Outcome
Carbonic anhydrase inhibitors	Acetazolamide	Chen et al. [43]	Retrospective	Acetazolamide may reduce macular cysts and thickness without visual acuity improvement.
Carotenoids	Zeaxanthin	Choi et al. [44]	Randomized control trial	Zeaxanthin provided no visual benefit or restoration of foveal macular pigment.
	Lutein, meso-zeaxanthin, zeaxanthin	Tan et al. [45]	Observational	Lutein, meso-zeaxanthin, and zeaxanthin supplements may stabilize vision and improve macular cavitations.
	AREDS2	Berger et al. [46]	Retrospective	Off-label AREDS2 may prevent anatomical and visual deterioration.
Laser	Argon laser photocoagulation	Stoffelns et al. [47]	Retrospective	Laser photocoagulation is not recommended due to a lack of visual improvement and the risk of subretinal neovascularization.
Photodynamic therapy (PDT)	PDT + intravitreal ranibizumab	Zehetner et al. [48]	Retrospective case series	Reduced-fluence PDT combined with intravitreal ranibizumab may help eyes with progressive vision loss.
	PTD	Hurley et al. [49]	Retrospective case series	No significant difference in best corrected visual acuity or central foveal thickness.
Intravitreal anti-VEGF	Bevacizumab	Matt et al. [50]	Interventional case series	Moderate overall effect, with some patients showing marked long-term functional and morphological benefit.
	Bevacizumab	Singler et al. [51]	Interventional case series	Ineffective in improving visual outcomes.
	Ranibizumab	Kupitz. et al. [52]	Case series	Monthly dosing for 1 year showed no long-term benefit 5 years after therapy.
	Ranibizumab	Do et al. [53]	Randomized controlled trial	Ranibizumab reduces leakage, but visual acuity improvement is minimal and similar to that of untreated eyes.
	Aflibercept	Bénichou et al. [54]	Case report	No improvement in anatomical or functional results.
Ciliary neurotrophic factor	NT-501	Chew et al. [39]	Phase 3, multicenter, randomized sham-controlled trials	NT-501 significantly reduced EZ area loss compared with sham treatment
Surgery	Pars plana vitrectomy with internal limiting membrane peeling	Singler et al. [51]	Interventional case series	Ineffective in improving visual outcomes.

#### 3.8.2. Proliferative MacTel Type 2

On the other hand, the proliferative phase is primarily managed with anti-VEGF agents, which have been shown to provide good functional and anatomical outcomes. A recent systematic review (2025) involving 377 eyes from 239 patients examined the effects of different anti-VEGF agents in both proliferative and non-proliferative MacTel cases. Half of the included studies reported anatomical and functional improvements with anti-VEGF therapy, while the remainder showed limited or no benefit. The most notable improvements were seen in patients with proliferative disease, compared with those in the non-proliferative stage. Nevertheless, the review emphasizes the need for further trials to establish evidence-based treatment guidelines [55].

In addition, a recent 2025 case report described the first use of intravitreal Faricimab to treat a MacTel patient with bilateral macular neovascularization. Following a total of three monthly bilateral injections, improvements were observed in both visual acuity and retinal structure, highlighting the potential role of anti-VEGF agents in managing the proliferative form of the disease [56].

Other treatment modalities described in the literature include argon laser photocoagulation, photodynamic therapy, transpupillary thermotherapy, and surgical excision of neovascular membranes. However, anti-VEGF therapy remains the first-line treatment option overall [7,57,58] (Table 3).

### 3.9. Present and Future Studies

In recent years, research in MacTel type 2 has expanded from mechanistic studies to coordinated observational and interventional investigations aimed at understanding disease progression, genetic and metabolic contributors, and potential therapeutic strategies. The ongoing MacTel natural history and registry study collects longitudinal clinical data from patients with confirmed MacTel Type 2, integrates genetic sequencing, and facilitates enrollment in future clinical trials, providing important insights into genotype–phenotype correlations and disease heterogeneity [64].

Complementing observational efforts, interventional studies are exploring metabolic modulation as a therapeutic strategy. The SAFE study, a Phase 2a trial, investigates whether systemic serine supplementation and fenofibrate can reduce circulating deoxysphingolipid levels, a key pathogenic metabolite identified through genetic and metabolomic studies [65]. Building on this, a Phase 3 randomized, placebo-controlled trial is evaluating long-term serine supplementation for slowing structural and functional retinal progression [66].

In parallel, neuroprotective approaches are being assessed. Phase 4 evaluations of encapsulated cell-based CNTF gene therapy (NT-501/ENCELTO) provide long-term safety and efficacy data, with trials examining the durability of therapeutic benefit in previously treated participants [67].

### 3.10. Prognosis and Progression

MacTel type 2 is a slowly progressive disease, which can have a stable visual acuity for a long time, while small paracentral scotomas may evolve, leading to changes in the paracentral visual field. Microperimetry is a more sensitive tool compared to visual acuity testing in monitoring the evolution of scotomas, especially when patients report low reading ability [18,68].

Marsonia et al. published in 2021 a study with 82 eyes of 47 patients diagnosed with MacTel, monitored a mean duration of 4.5 years (minimal 3 years, maximum 8.5 years). The mean logMAR best corrected visual acuity decreased from 0.25 ± 0.25 at baseline to 0.46 ± 0.42 by 4 years. After the fourth year, the vision in MacTel patients stabilized. Major causes of poor vision were choroidal neovascularization (active and scarred), foveal atrophy, and central pigmented plaques [69].

Regarding the stage of the disease, the proliferative form is associated with a reduced visual acuity compared to the non-proliferative form. Moreover, the involvement of the outer retinal layers is suggestive of a poorer visual prognosis [13].

## 4. Conclusions

Macular Telangiectasia Type 2 is a rare, progressive, bilateral retinal disorder primarily affecting individuals over 40 years of age. The disease can manifest in non-proliferative and proliferative stages, each with distinct structural changes detectable by optical coherence tomography. OCT provides critical information for early diagnosis and monitoring, often identifying retinal alterations before clinical signs are apparent. Therapeutic options remain limited, with interventions during the proliferative stage, particularly anti-VEGF therapy, offering the most favorable functional and anatomical outcomes. Emerging treatments, such as ciliary neurotrophic factor implants, show promise in slowing disease progression, highlighting the need for further research into stage-specific management strategies.

## Figures and Tables

**Table 1:** Common OCT features that were described in three studies. ELM = external limiting membrane.

Common OCT Feature	Study	Number of Eyes Involved (Percent from Total Eyes Examined)
Hyporeflective inner retinal cavities	Kim et al. [21]	121 (72.9%)
	Venkatesk et al. [16]	104 (49%)
Hyperreflective middle retinal layer	Venkatesk et al. [16]	184 (87%)
Hyporeflective outer retinal cavities	Venkatesk et al. [16]	32 (15%)
	Kim et al. [21]	Above ELM: 56 (33.7%) Below ELM: 52 (31.3%)
	Peto et al. [22]	243 (24.9%)
Outward bending of the inner retinal layers	Venkatesk et al. [16]	74 (35%)
Retinal pigment clumps		74 (35%)
Foveal contour irregularity		66 (31%)
Internal limiting membrane drape		62 (29%)
Discontinuity of the external limiting membrane	Kim et al. [21]	67 (40.4%)
Discontinuity of the ellipsoid zone	Kim et al. [21]	87 (52.4%)
	Peto et al. [22]	602 (61.8%)
Discontinuity of the interdigitation zone	Kim et al. [21]	94 (56.6%)

**Table 3:** Different treatment options and their outcomes for proliferative MacTel Type 2.

Treatment	Drug/Compound	Study	Type	Outcome
Laser	Argon laser photocoagulation	Reddy et al. [59]	Descriptive case report	Direct laser photocoagulation to right-angled vessels may be considered for proliferative type unresponsive to anti-VEGF.
	Transpupillary thermotherapy	Shukla et al. [60]	Non-randomized interventional case series	Transpupillary thermotherapy may be a safe and useful alternative treatment.
Photodynamic therapy (PDT)	PDT + intravitreal ranibizumab	Rishi et al. [61]	Case report	PDT combined with intravitreal ranibizumab appears effective for subretinal neovascular membrane.
Intravitreal anti-VEGF	Bevacizumab, ranibizumab, and aflibercept.	Karasu et al. [62]	Retrospective	Improve anatomical and visual outcomes; aflibercept and bevacizumab required fewer injections; ranibizumab and bevacizumab reduced subfoveal choroidal thickness, aflibercept does not.
	Bevacizumab and ranibizumab	Sen et al. [63]	Observational	Intravitreal bevacizumab and ranibizumab monotherapy both had similar efficacy.
	Ranibizumab and aflibercept	Çoban Karataş et al. [25]	Retrospective	Low vision may benefit from intravitreal anti-VEGF treatment.

## Data Availability

No new data were created or analyzed in this study.

## Abbreviations

MacTel = Macular Telangiectasia, OCT = Optical coherence tomography, CNTF = ciliary neurotrophic factor, anti-VEGF = anti-vascular endothelial growth factor, IJFT = Idiopathic juxtafoveolar retinal telangiectasias, RAV = right-angled vessels, OCTA = Optical coherence tomography angiography, FA = Fluorescein angiography, ILM = Internal limiting membrane, RCA = retinal–choroidal anastomoses, ELM = External limiting membrane, CMT = central macular thickness, EZ = ellipsoid zone, FAZ = foveal avascular zone, FAF = Fundus autofluorescence, PDT = photodynamic therapy.

## References

[1] Wu L. (2023). Unraveling the mysteries of macular telangiectasia 2: The intersection of philanthropy, multimodal imaging and molecular genetics. The 2022 founders lecture of the pan American vitreoretinal society. Int. J. Retin. Vitr..

[2] Gass J.D., Blodi B.A. (1993). Idiopathic juxtafoveolar retinal telangiectasis. Update of classification and follow-up study. Ophthalmology.

[3] Kedarisetti K.C., Narayanan R., Stewart M.W., Reddy Gurram N., Khanani A.M. (2022). Macular Telangiectasia Type 2: A Comprehensive Review. Clin. Ophthalmol..

[4] Klein R., Blodi B.A., Meuer S.M., Myers C.E., Chew E.Y., Klein B.E. (2010). The prevalence of macular telangiectasia type 2 in the Beaver Dam eye study. Am. J. Ophthalmol..

[5] Aung K.Z., Wickremasinghe S.S., Makeyeva G., Robman L., Guymer R.H. (2010). The prevalence estimates of macular telangiectasia type 2: The Melbourne Collaborative Cohort Study. Retina.

[6] Sallo F.B., Leung I., Mathenge W., Kyari F., Kuper H., Gilbert C.E., Bird A.C., Peto T., MacTel Study Group (2012). The prevalence of type 2 idiopathic macular telangiectasia in two African populations. Ophthalmic Epidemiol..

[7] Charbel Issa P., Gillies M.C., Chew E.Y., Bird A.C., Heeren T.F., Peto T., Holz F.G., Scholl H.P. (2013). Macular telangiectasia type 2. Prog. Retin. Eye Res..

[8] Scerri T.S., Quaglieri A., Cai C., Zernant J., Matsunami N., Baird L., Scheppke L., Bonelli R., Yannuzzi L.A., Friedlander M. (2017). Genome-wide analyses identify common variants associated with macular telangiectasia type 2. Nat. Genet..

[9] Bonelli R., Ansell B.R.E., Lotta L., Scerri T., Clemons T.E., Leung I., Peto T., Bird A.C., Sallo F.B., MacTel Consortium (2021). Genetic disruption of serine biosynthesis is a key driver of macular telangiectasia type 2 aetiology and progression. Genome Med..

[10] Kunčič A., Urbančič M., Dobovšek Divjak D., Hudler P., Debeljak N. (2025). Genetic Background of Macular Telangiectasia Type 2. Int. J. Mol. Sci..

[11] Yannuzzi L.A., Bardal A.M., Freund K.B., Chen K.J., Eandi C.M., Blodi B. (2006). Idiopathic macular telangiectasia. Arch. Ophthalmol..

[12] Tapia Quijada H.E., Mantolan Sarmiento C., Serrano García M., Betancor Caro N. (2020). Atypical bilateral presentation in idiopathic macular telangiectasia type 1. Arch. Soc. Esp. Oftalmol. (Engl. Ed.).

[13] Reddy N.G., Prabhu V., Sharma S.V., Acharya I., Mangla R., Yadav N.K., Chhablani J., Narayanan R., Venkatesh R. (2023). Baseline demographic, clinical and multimodal imaging features of young patients with type 2 macular telangiectasia. Int. J. Retin. Vitr..

[14] Clemons T.E., Gillies M.C., Chew E.Y., Bird A.C., Peto T., Figueroa M.J., Harrington M.W., MacTel Research Group (2010). Baseline characteristics of participants in the natural history study of macular telangiectasia (MacTel) MacTel Project Report No. 2. Ophthalmic Epidemiol..

[15] Chew E.Y., Peto T., Clemons T.E., Sallo F.B., Pauleikhoff D., Leung I., Jaffe G.J., Heeren T.F.C., Egan C.A., Charbel Issa P. (2022). Macular Telangiectasia Type 2: A Classification System Using MultiModal Imaging MacTel Project Report Number 10. Ophthalmol. Sci..

[16] Venkatesh R., Reddy N.G., Mishra P., Agrawal S., Mutalik D., Yadav N.K., Chhablani J. (2022). Spectral domain OCT features in type 2 macular telangiectasia (type 2 MacTel): Its relevance with clinical staging and visual acuity. Int. J. Retin. Vitr..

[17] Venkatesh R., Handa A., Chitturi S.P., Choudhary A., Prabhu V., Acharya I., Mangla R., Yadav N.K., Chhablani J. (2024). Right-angled vessel characteristics in different stages of type 2 macular telangiectasia (MacTel). Eye.

[18] Engelbert M., Chew E.Y., Yannuzzi L.A., Ryan S.J., Sadda S.R., Hinton D.R., Schachat A.P., Sadda S.R., Wilkinson C.P., Wiedemann P., Schachat A.P. (2013). Chapter 55—Macular Telangiectasia. Retina.

[19] Zerbinopoulos B., Goman-Baskin E., Greenberg P.B., Bryan R., Messina C. (2021). The Role of Diagnostic Imaging in Macular Telangiectasia Type 2. Fed. Pract..

[20] Venkatesh R., Nahata H., Reddy N.G., Mishra P., Mangla R., Yadav N.K., Chhablani J. (2023). Is Type 2 Macular Telangiectasia a Bilateral and Symmetrical Disease Entity?. J. Curr. Ophthalmol..

[21] Kim Y.H., Chung Y.R., Oh J., Kim S.W., Lee C.S., Yun C., Lee B., Ahn S.M., Choi E.Y., Jang S. (2020). Optical coherence tomographic features of macular telangiectasia type 2: Korean Macular Telangiectasia Type 2 Study-Report No. 1. Sci. Rep..

[22] Peto T., Heeren T.F.C., Clemons T.E., Sallo F.B., Leung I., Chew E.Y., Bird A.C. (2018). CORRELATION OF CLINICAL AND STRUCTURAL PROGRESSION WITH VISUAL ACUITY LOSS IN MACULAR TELANGIECTASIA TYPE 2: MacTel Project Report No. 6-The MacTel Research Group. Retina.

[23] Pandya B.U., Grinton M., Mandelcorn E.D., Felfeli T. (2024). RETINAL OPTICAL COHERENCE TOMOGRAPHY IMAGING BIOMARKERS: A Review of the Literature. Retina.

[24] Toto L., Di Antonio L., Mastropasqua R., Mattei P.A., Carpineto P., Borrelli E., Rispoli M., Lumbroso B., Mastropasqua L. (2016). Multimodal Imaging of Macular Telangiectasia Type 2: Focus on Vascular Changes Using Optical Coherence Tomography Angiography. Investig. Ophthalmol. Vis. Sci..

[25] Çoban Karataş M., Yılmaz G., Yüce Sezen A., Sarıtürk Ç. (2022). Clinical Features of Untreated Type 2 Macular Telangiectasia and Efficacy of Anti-Vascular Endothelial Growth Factor Therapy in Macular Neovascularization. Turk. J. Ophthalmol..

[26] Heeren T.F.C., Kitka D., Florea D., Clemons T.E., Chew E.Y., Bird A.C., Pauleikhoff D., Charbel Issa P., Holz F.G., Peto T. (2018). LONGITUDINAL CORRELATION OF ELLIPSOID ZONE LOSS AND FUNCTIONAL LOSS IN MACULAR TELANGIECTASIA TYPE 2. Retina.

[27] Cai C.X., Choong J., Farsiu S., Chiu S.J., Chew E.Y., Jaffe G.J. (2021). Retinal cavitations in macular telangiectasia type 2 (MacTel): Longitudinal structure-function correlations. Br. J. Ophthalmol..

[28] Batıoğlu F., Yanık Ö., Demirel S., Özmert E. (2023). Clinical Use of Optical Coherence Tomography Angiography in Retinal Diseases. Diagnostics.

[29] Zhang L., Van Dijk E.H.C., Borrelli E., Fragiotta S., Breazzano M.P. (2023). OCT and OCT Angiography Update: Clinical Application to Age-Related Macular Degeneration, Central Serous Chorioretinopathy, Macular Telangiectasia, and Diabetic Retinopathy. Diagnostics.

[30] Chandran K., Giridhar A., Gopalakrishnan M., Sivaprasad S. (2022). Microvascular changes precede visible neurodegeneration in fellow eyes of patients with asymmetric type 2 macular telangiectasia. Eye.

[31] Pauleikhoff L., Heeren T.F.C., Gliem M., Lim E., Pauleikhoff D., Holz F.G., Clemons T., Balaskas K., Egan C.A., MACTEL STUDY GROUP (2021). Fundus Autofluorescence Imaging in Macular Telangiectasia Type 2: MacTel Study Report Number 9. Am. J. Ophthalmol..

[32] Wu L. (2015). When is macular edema not macular edema? An update on macular telangiectasia type 2. Taiwan J. Ophthalmol..

[33] Van Romunde S.H.M., van der Sommen C.M., Martinez Ciriano J.P., Vingerling J.R., Yzer S. (2021). Prevalence and Severity of Diabetic Retinopathy in Patients with Macular Telangiectasia Type 2. Ophthalmol. Retin..

[34] Amram A.L., Whitmore S.S., Wang C., Clavell C., Lyons L.J., Rusakevich A.M., Han I., Folk J., Boldt H.C., Stone E.M. (2025). Progressive inner retinal neurodegeneration in non-proliferative macular telangiectasia type 2. Br. J. Ophthalmol..

[35] Dorrell M.I., Aguilar E., Jacobson R., Yanes O., Gariano R., Heckenlively J., Banin E., Ramirez G.A., Gasmi M., Bird A. (2009). Antioxidant or neurotrophic factor treatment preserves function in a mouse model of neovascularization-associated oxidative stress. J. Clin. Investig..

[36] Chew E.Y., Clemons T.E., Peto T., Sallo F.B., Ingerman A., Tao W., Singerman L., Schwartz S.D., Peachey N.S., Bird A.C. (2015). Ciliary neurotrophic factor for macular telangiectasia type 2: Results from a phase 1 safety trial. Am. J. Ophthalmol..

[37] Kauper K., McGovern C., Sherman S., Heatherton P., Rapoza R., Stabila P., Dean B., Lee A., Borges S., Bouchard B. (2012). Two-year intraocular delivery of ciliary neurotrophic factor by encapsulated cell technology implants in patients with chronic retinal degenerative diseases. Investig. Ophthalmol. Vis. Sci..

[38] Chew E.Y., Clemons T.E., Jaffe G.J., Johnson C.A., Farsiu S., Lad E.M., Guymer R., Rosenfeld P., Hubschman J.P., Constable I. (2019). Effect of Ciliary Neurotrophic Factor on Retinal Neurodegeneration in Patients with Macular Telangiectasia Type 2: A Randomized Clinical Trial. Ophthalmology.

[39] Chew E.Y., Gillies M., Jaffe G.J., Gaudric A., Egan C., Constable I., Clemons T., Aaberg T., Manning D.C., Hohman T.C. (2025). Cell-Based Ciliary Neurotrophic Factor Therapy for Macular Telangiectasia Type 2. NEJM Evid..

[40] Hoy S.M. (2025). Revakinagene Taroretcel: First Approval. Mol. Diagn. Ther..

[41] Nystuen A., Gonzalez-Lopez E., Kauper K.A., Eade K., Aaberg T.M. (2025). Neuroprotective properties of ciliary neurotrophic factor in the retina for the treatment of macular telangiectasia type 2. Cytokine Growth Factor Rev..

[42] Neurotech Pharmaceuticals, Inc. (2025). ENCELTO (Revakinagene Taroretcel-Lwey) Implant, for Intravitreal Use: US Prescribing Information. https://www.fda.gov/.

[43] Chen J.J., Sohn E.H., Folk J.C., Mahajan V.B., Kay C.N., Boldt H.C., Russell S.R. (2014). Decreased macular thickness in nonproliferative macular telangiectasia type 2 with oral carbonic anhydrase inhibitors. Retina.

[44] Choi R.Y., Gorusupudi A., Wegner K., Sharifzadeh M., Gellermann W., Bernstein P.S. (2017). MACULAR PIGMENT DISTRIBUTION RESPONSES TO HIGH-DOSE ZEAXANTHIN SUPPLEMENTATION IN PATIENTS WITH MACULAR TELANGIECTASIA TYPE 2. Retina.

[45] Tan A.C., Balaratnasingam C., Yannuzzi L.A. (2016). Treatment of Macular Telangiectasia Type 2 with Carotenoid Supplements Containing Meso-Zeaxanthin: A Pilot Study. Ophthalmic Surg. Lasers Imaging Retin..

[46] Berger T.A., Manry M.W., Lindsell L.B., Osher J.M., Miller D.M., Foster R.E., Riemann C.D., Petersen M.R., Sisk R.A. (2021). Outcome of Off-Label AREDS 2 Supplementation for the Treatment of Macular Degeneration in Non-Proliferative Idiopathic Type 2 Macular Telangiectasia. Clin. Ophthalmol..

[47] Stoffelns B.M., Schoepfer K., Kramann C. (2010). Idiopathische makuläre Teleangiektasie (IMT)—Verlaufsbeobachtung mit und ohne Laserphotokoagulation [Idiopathic macular telangiectasia—Follow-up with and without laser photocoagulation]. Klin. Monbl. Augenheilkd..

[48] Zehetner C., Haas G., Treiblmayr B., Kieselbach G.F., Kralinger M.T. (2013). Reduced-fluence photodynamic therapy combined with ranibizumab for nonproliferative macular telangiectasia type 2. Ophthalmologica.

[49] Hurley D.J., Gallagher D., Petronzi V., O’Rourke M., Kinsella F., Townley D. (2023). Examining the efficacy of verteporfin photo-dynamic therapy (PDT) at different dose & fluence levels. Photodiagnosis Photodyn. Ther..

[50] Matt G., Sacu S., Ahlers C., Schütze C., Dunavoelgyi R., Prager F., Pruente C., Schmidt-Erfurth U. (2010). Thirty-month follow-up after intravitreal bevacizumab in progressive idiopathic macular telangiectasia type 2. Eye.

[51] Sigler E.J., Randolph J.C., Calzada J.I., Charles S. (2013). Comparison of observation, intravitreal bevacizumab, or pars plana vitrectomy for non-proliferative type 2 idiopathic macular telangiectasia. Graefe’s Arch. Clin. Exp. Ophthalmol..

[52] Kupitz E.H., Heeren T.F., Holz F.G., Charbel Issa P. (2015). POOR LONG-TERM OUTCOME OF ANTI-VASCULAR ENDOTHELIAL GROWTH FACTOR THERAPY IN NONPROLIFERATIVE MACULAR TELANGIECTASIA TYPE 2. Retina.

[53] Do D.V., Bressler S.B., Cassard S.D., Gower E.W., Tabandeh H., Jefferys J.L., Bressler N.M. (2014). Ranibizumab for macular telangiectasia type 2 in the absence of subretinal neovascularization. Retina.

[54] Bénichou J., Soler V., Denis D., Matonti F. (2017). Inefficacité de l’aflibercept dans le traitement des télangiectasies maculaires idiopathiques de type 2 sans néovascularisation [Inefficacy of aflibercept in the treatment of idiopathic macular telangiectasia type 2 without neovascularization]. J. Fr. Ophtalmol..

[55] Sriranganathan A., Grad J., Mihalache A., Popovic M.M., Kertes P.J., Kohly R., Muni R.H. (2025). Anti-Vascular Endothelial Growth Factor Treatment Outcomes in Macular Telangiectasia: A Systematic Review. Ophthalmologica.

[56] Rao P., Rao A., Jaffer J.M., John A.S. (2025). Intravitreal Faricimab for the Management of Bilateral Macular Neovascularization Secondary to Macular Telangiectasia Type 2. Cureus.

[57] Chatziralli I.P., Sharma P.K., Sivaprasad S. (2017). Treatment Modalities for Idiopathic Macular Telangiectasia: An Evidence-Based Systematic Review of the Literature. Semin. Ophthalmol..

[58] Khodabande A., Roohipoor R., Zamani J., Mirghorbani M., Zolfaghari H., Karami S., Modjtahedi B.S. (2019). Management of Idiopathic Macular Telangiectasia Type 2. Ophthalmol. Ther..

[59] Reddy N.G., Venkatesh R., Jayadev C., Agrawal S., Yadav N.K., Chhablani J. (2023). DIRECT LASER PHOTOCOAGULATION TO THE DILATED RIGHT-ANGLED VESSEL IN THE MANAGEMENT OF PROLIFERATIVE TYPE 2 MACULAR TELANGIECTASIA. Retin. Cases Brief. Rep..

[60] Shukla D., Singh J., Kolluru C.M., Kim R., Namperumalsamy P. (2004). Transpupillary thermotherapy for subfoveal neovascularization secondary to group 2A idiopathic juxtafoveolar telangiectasis. Am. J. Ophthalmol..

[61] Rishi P., Shroff D., Rishi E. (2008). Combined photodynamic therapy and intravitreal ranibizumab as primary treatment for subretinal neovascular membrane (SRNVM) associated with type 2 idiopathic macular telangiectasia. Graefe’s Arch. Clin. Exp. Ophthalmol..

[62] Karasu B., Gunay B.O. (2020). Comparison of anatomical and visual outcomes following different anti-vascular endothelial growth factor treatments in subretinal neovascular membrane secondary to type 2 proliferative macular telangiectasia. Graefe’s Arch. Clin. Exp. Ophthalmol..

[63] Sen S., Rajan R.P., Damodaran S., Arumugam K.K., Kannan N.B., Ramasamy K. (2021). Real-world outcomes of intravitreal anti-vascular endothelial growth factor monotherapy in proliferative type 2 macular telangiectasia. Graefe’s Arch. Clin. Exp. Ophthalmol..

[64] A Natural History Observation and Registry Study of Macular Telangiectasia Type 2: The MacTel Study, Mayo Clinic Clinical Trials. https://www.mayo.edu/research/clinical-trials/cls-20366168.

[65] Serine and Fenofibrate Study in Patients with MacTel Type 2 (SAFE), Clinical Trials Registry. https://ichgcp.net/clinical-trials-registry/NCT04907084.

[66] Study of Serine Supplementation to Protect Vision in MacTel (SEErine). https://clinicaltrials.gov/study/NCT07342439?term=TREATMENt.

[67] Phase 4 Study: Long-Term Safety and Efficacy of NT-501 in MacTel Type 2, Including Sham Procedure Participants. https://clinicaltrials.gov/study/NCT06971939.

[68] Heeren T.F., Clemons T., Scholl H.P., Bird A.C., Holz F.G., Charbel Issa P. (2015). Progression of Vision Loss in Macular Telangiectasia Type 2. Investig. Ophthalmol. Vis. Sci..

[69] Marsonia K., Kiran Chandra K., Ali M.H., Chhablani J., Narayanan R. (2022). Long term follow-up of visual acuity and incidence of subretinal neovascularization in Mactel Type 2 in 82 Eyes. Semin. Ophthalmol..

